# Supplementary material for: Accurate Blood-Based Diagnostic Biosignatures for Alzheimer’s Disease via Automated Machine Learning
Source: J Clin Med. 2020 Sep 18;9(9):3016. doi: 10.3390/jcm9093016 (PMC7563988; doi:10.3390/jcm9093016)
Supplement: Supplementary file 1 [file jcm-09-03016-s001.pdf]

Supplementary material

Supplementary figures

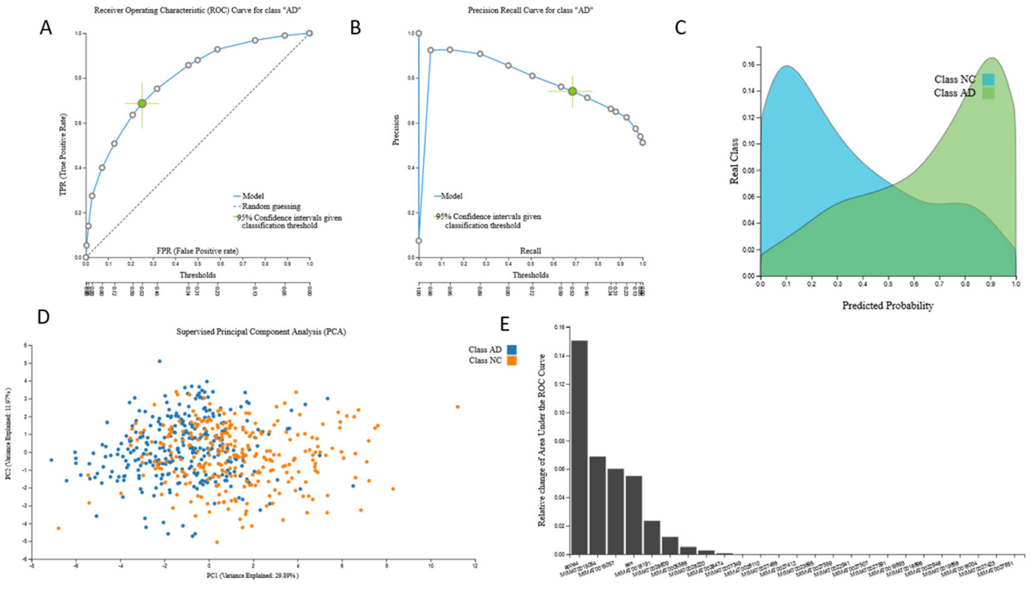

Supplementary Figure 1. **2,566-features miRNA-transcriptomic dataset signature performance, selected predictors and model inspection**: A. Receiver Operating Characteristic (ROC) Curve for AD class, B. Precision-recall plot, C. Probabilities density plot, D. Supervised Principal Component Analysis plot depicting discrimination between AD patients and age-sex matched cognitively healthy individuals, E. Cumulative feature importance plot of the 25 miRNA predictors of the signature.

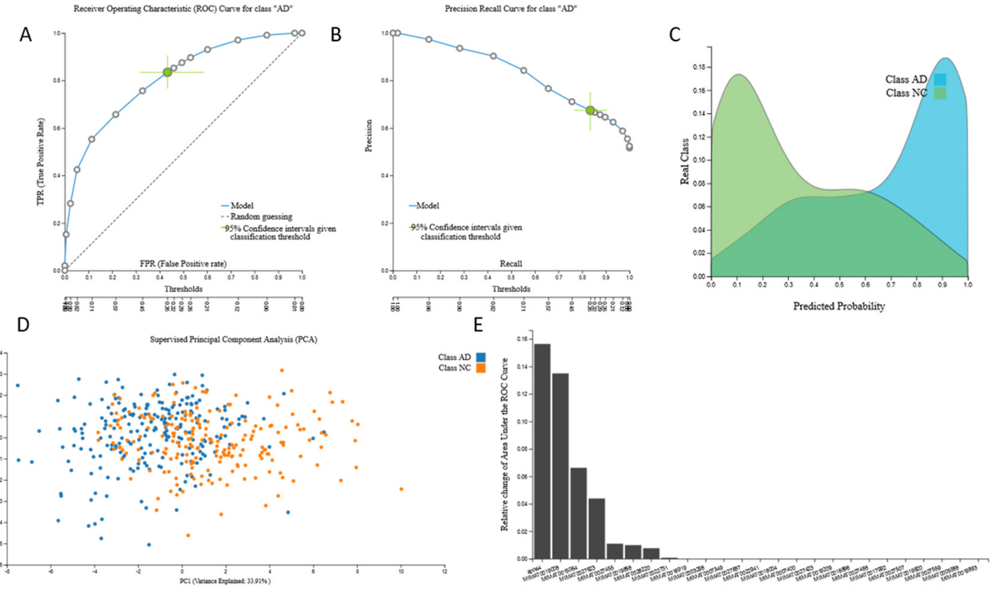

Supplementary Figure 2. **2,566-features miRNA-transcriptomic sub-dataset (train) signature performance, selected predictors and model inspection**: A. Receiver Operating Characteristic (ROC) Curve for AD class, B. Precision-recall plot, C. Probabilities density plot, D. Supervised Principal Component Analysis plot depicting discrimination between AD patients and age-sex matched cognitively healthy individuals, E. Cumulative feature importance plot of the 25 miRNA predictors of the signature.

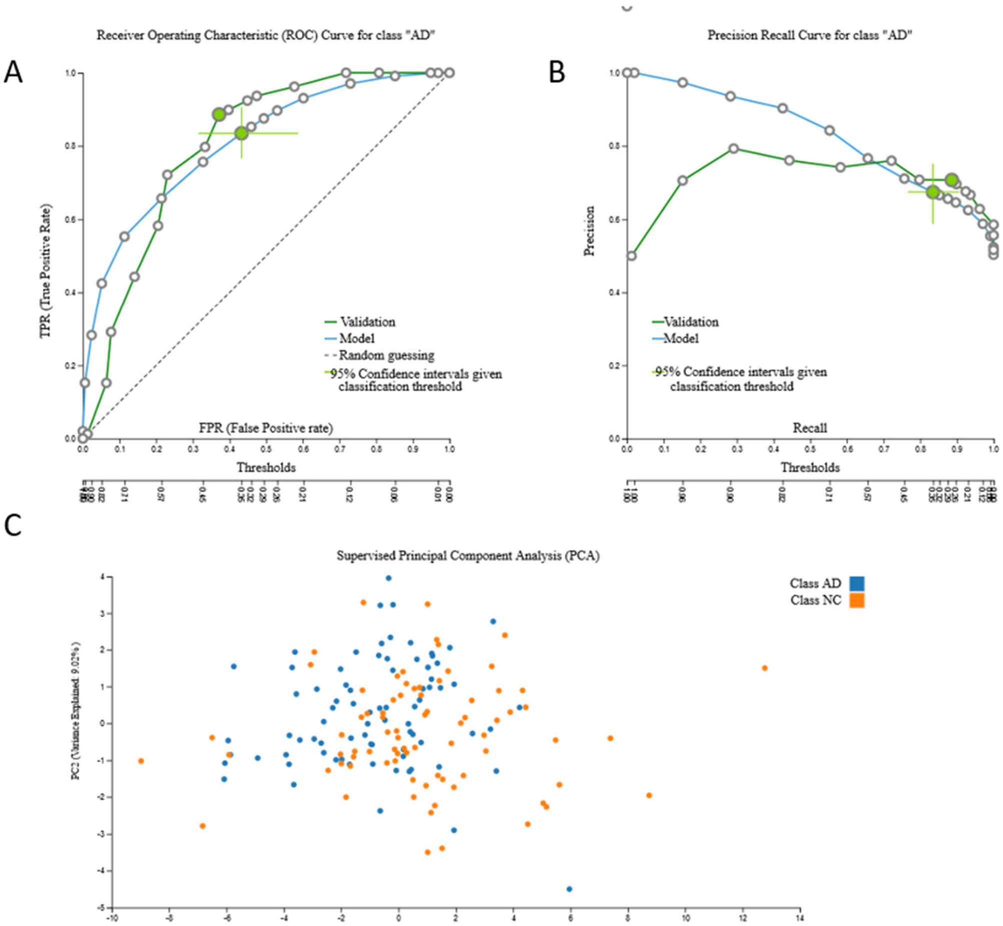

Supplementary Figure 3. **Validation of 2,566-features miRNA sub-dataset (test) signature performance**: A. Receiver Operating Characteristic (ROC) Curve for AD class, B. Precision-recall plot, C. Supervised Principal Component Analysis plot depicting discrimination between AD patients and age-sex matched cognitively healthy individuals in test dataset.

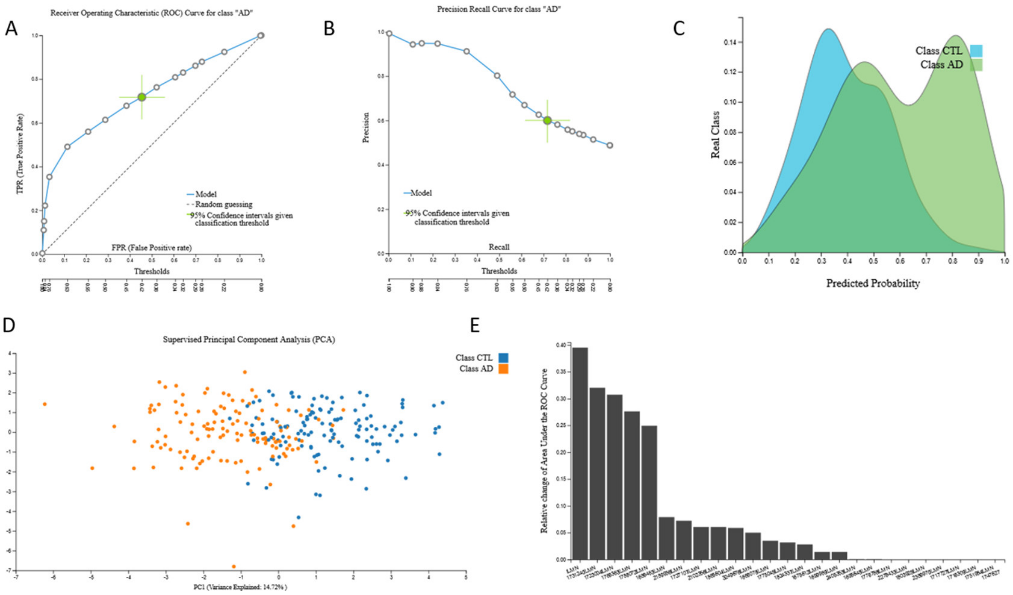

Supplementary Figure 4. **32,053-features mRNA-transcriptomic dataset signature performance, selected predictors and model inspection**: A. Receiver Operating Characteristic (ROC) Curve for AD class, B. Precision-recall plot, C. Probabilities density plot, D. Supervised Principal Component Analysis plot depicting discrimination between AD patients and age-sex matched cognitively healthy individuals, E. Cumulative feature importance plot of the 25 mRNA predictors of the reference signature.

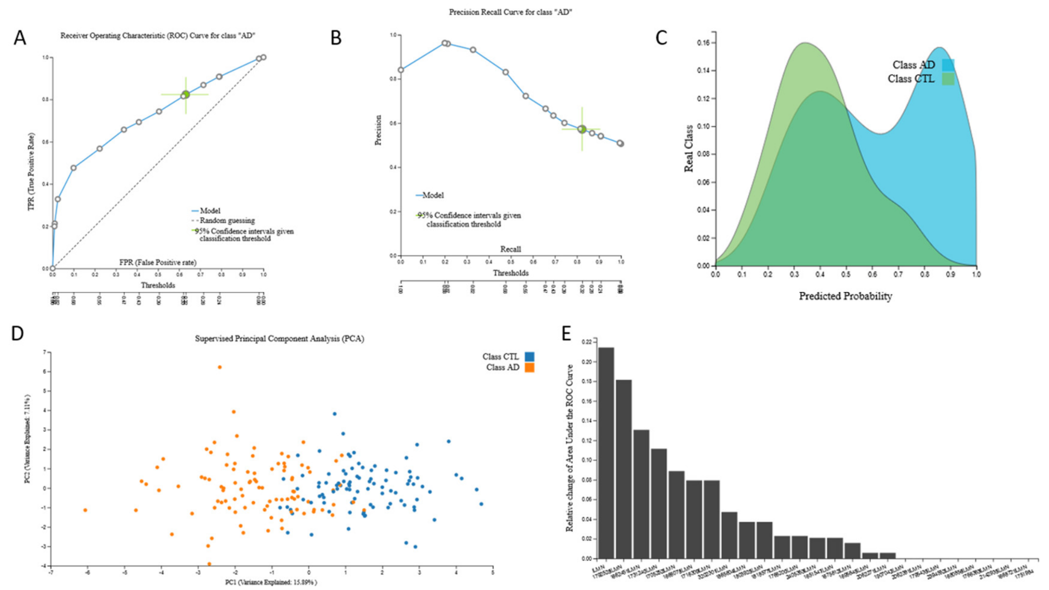

Supplementary Figure 5. **32,053-features mRNA-transcriptomic sub-dataset (train) signature performance, selected predictors and model inspection**: A. Receiver Operating Characteristic (ROC) Curve for AD class, B. Precision-recall plot, C. Probabilities density plot, D. Supervised Principal Component Analysis plot depicting discrimination between AD patients and age-sex matched cognitively healthy individuals, E. Cumulative feature importance plot of the 25 mRNA predictors of the reference signature.

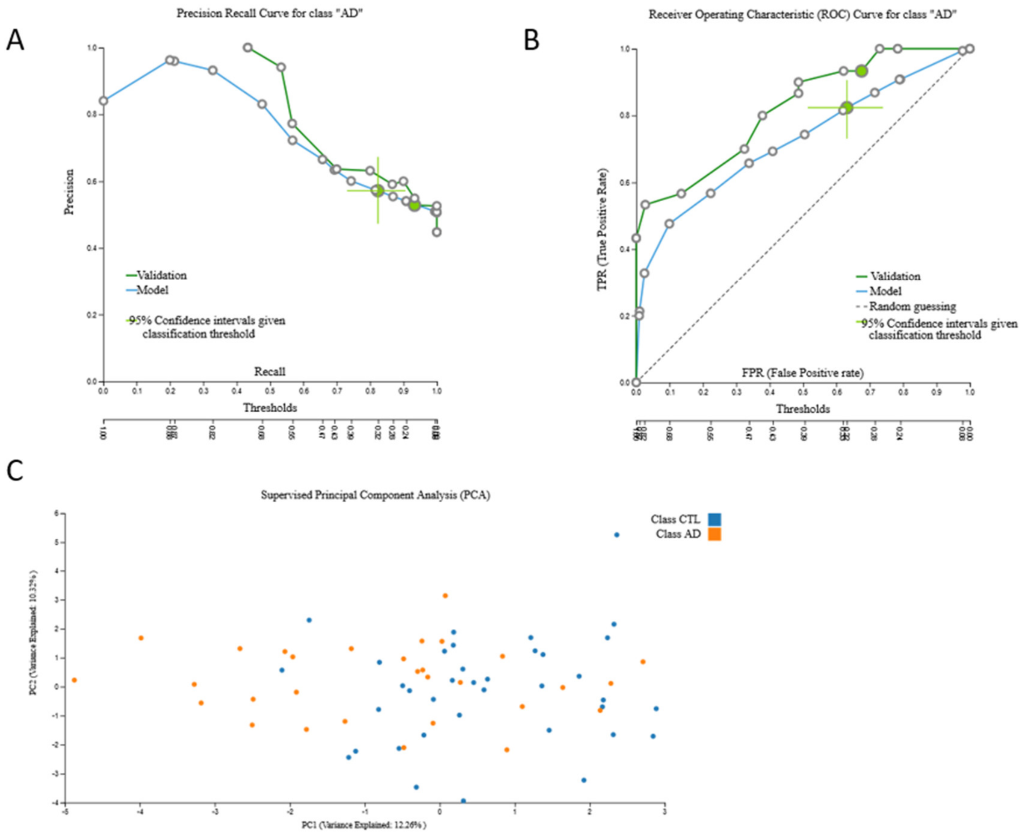

Supplementary figure 6. Validation of 32,053-features mRNA sub-dataset (test) signature performance: A. Receiver Operating Characteristic (ROC) Curve for AD class, B. Precision-recall plot, C. Supervised Principal Component Analysis plot depicting discrimination between AD patients and age-sex matched cognitively healthy individuals in test dataset.

Metric	Mean estimate	95% confidence interval	Unadjusted estimate	Base line	Statistically significant
Accuracy	0.929	[0.846, 1.000]	0.929	0.686	✓
Balanced Accuracy	0.925	[0.825, 1.000]	0.923	0.500	✓
F1 Score	0.926	[0.848, 1.000]	0.929	0.814	✓
Precision	0.900	[0.789, 1.000]	0.902	0.686	✓
True Positive Rate	0.957	[0.875, 1.000]	0.958	1.000	—
Specificity	0.774	[0.500, 1.000]	0.773	0.000	✓
True Positives (TP)	0.651	[0.500, 0.800]	0.657	0.686	—
True Negatives (TN)	0.247	[0.120, 0.385]	0.243	0.000	✓
False Positives (FP)	0.072	[0.000, 0.154]	0.071	0.314	✓
False Negatives (FN)	0.029	[0.000, 0.083]	0.029	0.000	—
Average F1 Score	0.907	[0.812, 0.988]	0.909	NaN	—

Supplementary figure 7. Performance metrics when classification threshold (0.440) is optimized for best Accuracy.

Metric	Mean estimate	95% confidence interval	Unadjusted estimate	Base line	Statistically significant
Accuracy	0.695	[0.550, 0.840]	0.700	0.686	—
Balanced Accuracy	0.522	[0.500, 0.600]	0.523	0.500	—
F1 Score	0.578	[0.364, 0.759]	0.588	NaN	—
Precision	1.000	[1.000, 1.000]	1.000	NaN	—
True Positive Rate	0.413	[0.222, 0.611]	0.417	0.000	✓
Specificity	1.000	[1.000, 1.000]	1.000	1.000	—
True Positives (TP)	0.281	[0.143, 0.429]	0.286	0.000	✓
True Negatives (TN)	0.319	[0.174, 0.462]	0.314	0.314	—
False Positives (FP)	0.000	[0.000, 0.000]	0.000	0.000	—
False Negatives (FN)	0.400	[0.240, 0.545]	0.400	0.686	✓
Average F1 Score	0.395	[0.277, 0.503]	0.338	NaN	—

Supplementary figure 8. Performance metrics when classification threshold (0.981) is optimized for best Specificity.

Metric	Mean estimate	95% confidence interval	Unadjusted estimate	Base line	Statistically significant
Accuracy	0.901	[0.808, 1.000]	0.900	0.314	✓
Balanced Accuracy	0.927	[0.861, 1.000]	0.927	0.500	✓
F1 Score	0.894	[0.810, 0.971]	0.897	0.814	—
Precision	0.811	[0.680, 0.944]	0.814	0.686	—
True Positive Rate	1.000	[1.000, 1.000]	1.000	1.000	—
Specificity	0.502	[0.200, 0.800]	0.500	0.000	✓
True Positives (TP)	0.681	[0.538, 0.826]	0.686	0.686	—
True Negatives (TN)	0.160	[0.042, 0.280]	0.157	0.000	✓
False Positives (FP)	0.159	[0.045, 0.286]	0.157	0.314	✓
False Negatives (FN)	0.000	[0.000, 0.000]	0.000	0.000	—
Average F1 Score	0.878	[0.810, 0.942]	0.880	0.646	✓

Supplementary figure 9. Performance metrics when classification threshold (0.197) is optimized for best True positive rate.

Supplementary Tables

Supplementary Table 1 Available information about the patients/cases in the datasets used regarding age and sex.

Dataset		Alzheimer's disease samples	Cognitively healthy samples
Metabolomic 1	Mean Age (y)	Non available	Non available
	Gender		
Metabolomic 2	Mean Age (y)	79	82
	Gender	4 female/14 male	5 female/16 male
Proteomic	Mean Age (y)	65	69
	Gender	16 female/10 male	15 female/22 male
Transcriptomic 1	Mean Age (y)	70	67
	Gender	25 female/23 male	11 female/11 male
Transcriptomic 2	Mean Age (y)	72	72
	Gender	190 female/110 male	137 female/152 male
Transcriptomic 3	Mean Age (y)	75	73
	Gender	91 female/43 male	59 female/41 male
Transcriptomic 4	Mean Age (y)	77	76
	Gender	75 female/51 male	78 female/53 male

Supplementary Table 2 Results of the GeneCard search of the proteins selected in the biosignatures in relation to AD.

JADBIO protein predictors	Description	Related pathway	Alzheimer Rank/Score*
LRRIQ2	Leucine Rich Repeats and IQ Motif Containing Protein 2	Organelle biogenesis and maintenance and Regulation of PLK1 Activity at G2/M Transition	NF
CAMLG	Calcium signal-modulating cyclophilin ligand	RANK Signaling in Osteoclasts and Ca, cAMP and Lipid Signaling	NF
IL4	interleukin 4	Immune response Fc epsilon RI pathway and PEDF Induced Signaling	551/6.72
TPM1	tropomyosin 1	Dilated cardiomyopathy (DCM) and Cardiac muscle contraction	2014/1.43
IL20	interleukin 20	PEDF Induced Signaling and Akt Signaling	3562/0.78
DIABLO	diablo homolog (Drosophila	Apoptosis Modulation and Signaling and Apoptosis and survival. Regulation of Apoptosis by Mitochondrial Proteins	6986/0.40
VRK3	Serine/threonine-protein kinase	Nuclear Events (kinase and transcription factor activation) and Signaling by GPCR	7550/0.36

*According to GeneCards list about AD (https://www.genecards.org/Search/Keyword?queryString=Alzheimer); NF: Not Found in the GeneCards list

Supplementary Table 3 Results of the GeneCard search of the miRNAs selected in the biosignatures in relation to AD.

JADBIO miRNA predictors	MiRNA Predicted targets*	Related pathway	Alzheimer Rank/Score**
hsa-miR-30d-5p	1539	miRNA targets in ECM and membrane receptors and MicroRNAs in cancer	1298/2.45
hsa-miR-29c-3p	1034	MicroRNAs in cancer and Metastatic brain tumor.	915/3.67
Brain-mir-182	388	Alzheimer's Disease	1398/2.10

*According to miRbase (http://www.mirbase.org/), **According to GeneCards list about AD (https://www.genecards.org/Search/Keyword?queryString=Alzheimer); NF: Not Found in the GeneCards list

Supplementary Table 4 Results of the GeneCard search of the mRNAs selected in the biosignatures in relation to AD.

JADBIO mRNA predictors (PROBE id)	Gene	Description	Related pathway	Alzheimer Rank/Score*
ILMN_2097421	MRPL51	mitochondrial ribosomal protein L51	Organelle biogenesis and maintenance and Mitochondrial translation	4642/0.74
ILMN_1699829	CTGF	connective tissue growth factor	NF-KappaB Family Pathway and amb2 Integrin signaling.	NF
ILMN_1795055	LRRC3	leucine rich repeat containing 3	N/A	NF
ILMN_1789436	DENND1B	DENN Domain Containing 1B	RAB GEFs exchange GTP for GDP on RABs and Vesicle-mediated transport.	NF
ILMN_1716498	LOC651966	N/A	N/A	N/A
ILMN_1768856	CHAT	Choline O-Acetyltransferase	Neurotransmitter Release Cycle and Transmission across Chemical Synapses.	55/47
ILMN_1860308	NBEAL1	neurobeachin like 1	N/A	NF
ILMN_1678325	LOC642335	N/A	N/A	
ILMN_1670217	MCF2L2	MCF.2 Cell Line Derived Transforming Sequence-Like 2	N/A	NF
ILMN_1671516	CIR	Corepressor Interacting With RBPJ	Development NOTCH1-mediated pathway for NF-KB activity modulation	NF
ILMN_1789618	FER	FER Tyrosine Kinase	Signaling by GPCR and Immune response Fc epsilon RI pathway.	NF
ILMN_1739297	GALNT4	Polypeptide N-Acetylgalactosaminyltransferase 4	Mucin type O-glycan biosynthesis and Metabolism of proteins	NF
ILMN_1676670	TMED8	Transmembrane P24 Trafficking Protein Family Member 8	N/A	NF
ILMN_1897103	N/A	N/A	N/A	N/A
ILMN_1882018	N/A	N/A	N/A	N/A
ILMN_2308903	WFDC3	WAP Four-Disulfide Core Domain 3	Serine-type endopeptidase inhibitor activity and peptidase inhibitor activity.	NF
ILMN_1884878	N/A	N/A	N/A	N/A
ILMN_1747241	IWS1	Interacts With SUPT6H, CTD Assembly Factor 1	Gene Expression and HIV Transcription Elongation.	2789/0.97
ILMN_1817714	N/A	N/A	N/A	N/A
ILMN_1791304	LOC651493	N/A	N/A	N/A
ILMN_1896943	N/A	N/A	N/A	N/A

ILMN_1711120	ARC	Activity Regulated Cytoskeleton Associated Protein	Neuroscience and PEDF Induced Signaling	NF
ILMN_1693183	SORBS2	Sorbin And SH3 Domain Containing 2	Structural constituent of cytoskeleton	4322/0.74
ILMN_1695868	PRICKLE4	Prickle Planar Cell Polarity Protein 4	Wnt signaling pathway	NF
ILMN_1730491	FMNL2	Formin Like 2	Signaling by GPCR and Signaling by Rho GTPases	7680/0.36

*According to GeneCards list about AD (https://www.genecards.org/Search/Keyword?queryString=Alzheimer); NF: Not Found in the GeneCards list, N/A: Non-Applicable
